# Monitoring and early warning of a metal mine tailings pond based on a deep learning bidirectional recurrent long and short memory network

**DOI:** 10.1371/journal.pone.0273073

**Published:** 2022-10-13

**Authors:** Zhanjie Jing, Xiaohong Gao

**Affiliations:** 1 Department of Geographic Information Science, Qinghai Normal University, Xining, China; 2 Institute of Resources and Ecology, Yili Normal University, Yining, Yili Kazakh Autonomous Prefecture, China; 3 College of Biology and Geography Sciences, Yili Normal University, Yining, Yili Kazakh Autonomous Prefecture, China; 4 Qinghai Province Key Laboratory of Physical Geography and Environmental Process, College of Geographical Science, Qinghai Normal University; University of Bonab, ISLAMIC REPUBLIC OF IRAN

## Abstract

The effective monitoring and early warning capability of metal mine tailings ponds can improve the associated safety risk management level. The infiltration line is an important core index of tailings pond stability. In this paper, a tailings pond monitoring and early warning system, which provides technical support for the design and daily management of tailings reservoir early warning systems, is constructed. Based on a deep learning bidirectional recurrent long and short memory network, an infiltration line prediction model with univariate input and an infiltration line prediction model with multivariate input are proposed. The data adopted are those from four monitoring points of the same cross-section at different positions and data from one adjacent internal lateral displacement and internal vertical displacement monitoring point. Using the adaptive moment estimation (Adam) optimization algorithm and the root mean square error (RMSE) model evaluation metric, the multilayer perceptron model, univariate input model, and multivariate input model are compared. This work shows that their RMSEs are 0.10611, 0.09966, and 0.11955, respectively.

## Introduction

Mineral resources are an important material basis for promoting economic and social development and an indispensable support for ensuring social stability and sustainable economic development. In the process of mining mineral resources, the treatment of tailings should avoid causing environmental problems, and at the same time, the redevelopment and utilization of tailings resources should be considered. The solid-liquid mixed storehouse formed by the concentrated storage of mineral waste residues is called a tailings pond [1]. Many serious accidents related to tailings ponds have occurred worldwide. The basic causes of tailings dam failure can be divided into flood overtopping, dam cracks, seepage damage and dam landslides. However, the failure of tailings dams is often caused by many factors, which are essentially due to the influence of the external environment, such as the increased load through tailings dams, earthquakes, rainfall, floods and dam foundation settlement [2]. The stress field and seepage field of the tailings reservoir change, which leads to instability of the dam body. The infiltration line of the seepage field in tailings ponds is called the "lifeline" of tailings ponds, and the determination of the seepage field is the basis of studying dam failure in tailings ponds. The position of the infiltration line affects the stability of the dam slope [3]. The consolidation speed of tailings below the infiltration line is slow, and tailings close to saturation increase the weight of the dam body, thus reducing the shear strength and effective stress of the dam body. In addition, rainstorms, floods and drainage facility failures usually lead to an increase in the saturation line in tailings dams, which leads to seepage damage. For tailings ponds, when the deformation conditions caused by seepage are met, a piping effect occurs in tailings dams. The material properties of tailings change after the piping effect, which leads to an increase in permeability and a decrease in shear strength and deformation modulus. Eventually, the tailings pond collapses, and the tailings dam breaks. Because of the complicated geological conditions and inaccurate boundary conditions of tailings ponds, it is difficult to find the exact solution of the seepage field and stress field of tailings ponds through theoretical research.

Li et al. [4] evaluated tailings reservoir disasters by the dynamic hierarchical gray relational analysis method and established an evaluation index and dynamic early warning index. Li et al. [5] studied the safety monitoring and early warning of tailings by examining the spatial evolution process of sediment flow and simulated the dam failure process of tailings dams in three-dimensional space. Wang et al. [6] designed and implemented a tailings pond monitoring system based on the Internet of Things and realized the real-time collection of monitoring data. Recently, an increasing number of researchers have applied data-driven methods in risk prediction, such as the artificial neural network (ANN). Through machine learning, we can use more data information, including nonlinear, mutual relations, and even hidden information in imperceptible data. Through model training, the safety trend of tailings ponds can be predicted, and the safety assessment and risk prediction performance of tailings ponds can be greatly improved. With the development of 5G networks, artificial intelligence, big data and other technologies, industrial production and processing have become more intelligent. Increasingly more monitoring data are being collected. It is very difficult to automatically process a large amount of data through a shallow network processing method.

The deep learning method has a strong feature extraction performance. In the first stage, it reduces the requirement of data feature description and provides a new solution for processing massive data. Deep learning methods have achieved great breakthroughs and have been used as advanced models in the field of artificial intelligence (AI) [7], such as for image semantic analysis and recognition [8,9], natural language processing [10] and speech recognition [11]. Deep learning has been widely used in various fields, such as earthquake and emergency response [12–15], biomedicine [16–18], mechanical fault diagnosis management [19–21], public transportation [22,23], energy [24–26], novel electrodes for future devices [27], and tailings dam failure and risk management [28–31]. In deep learning, the bidirectional long and short memory network model can combine the historical state and current memory to address time series problems. It is mainly used to describe the relationship between current data and previous input data, and its memory is used to save the internal information of previous data. The bidirectional cyclic long-short memory network model can not only learn the shallow nonlinear network structure but also approximate complex functions, extract the essential features of input time series data, and remember information for a long time. On this basis, the following topics are studied in this paper:

1. A monitoring and early warning system is constructed for tailings ponds that integrates a deep learning bidirectional cyclic long and short memory network.
2. Based on a deep learning bidirectional recurrent long-short memory network, a tailings pond infiltration line prediction model is proposed that has a single-variable input and a multi-variable input.
3. Through experiments, the multilayer perceptron model is compared with the model based on a bidirectional recurrent long short-term memory network.

## Materials and methods

### Deep learning bidirectional recurrent long and short memory network

**Fig 1:**
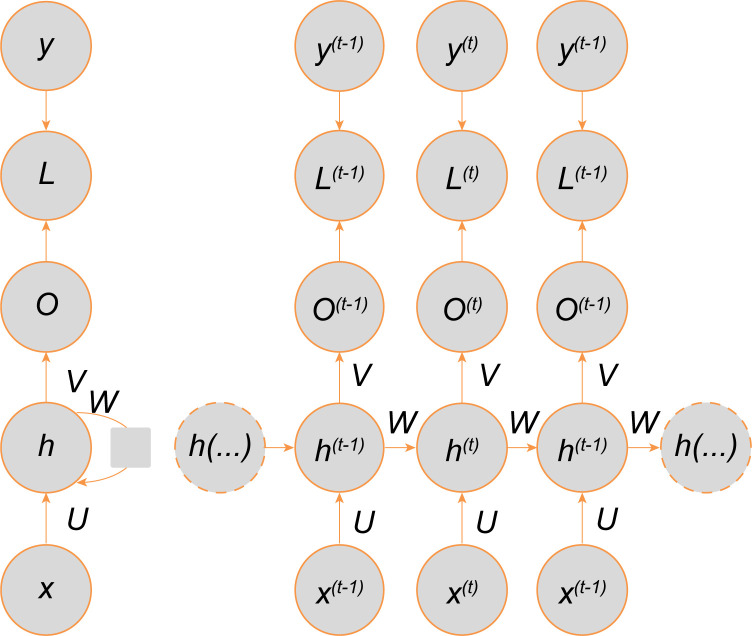
The computational graph to compute the training loss of the recurrent network.

A recurrent neural network is the foundation of a bidirectional cyclic long and short memory network. The calculation of the cyclic neural network forms a directed graph, and the expansion calculation diagram of the training loss of the basic cyclic neural network is shown in Fig 1. For time step t, h is the hidden state of the cyclic neural network, x is the input time series vector, and y^ is the output vector of the cyclic neural network mapping the input vector of the x value. L is a measure of the loss function between each model output y^ and the corresponding training target y. Comparing it with the target y, the bias vectors b and c and the weight matrices U, W, and V are the links from the input to the hidden layer, the hidden layer to the hidden layer, and the hidden layer to the output layer, respectively. Its calculation expression is as follows: (1)–(3), where θ is the parameter learning function with minimum model loss and f is the nonlinear activation function.


$$h_{t}=f(Uh_{t}+Wx_{t}+b)$$ (1)

$$y^{⋀(t)}=Vh_{t}+c$$ (2)

$$L=\theta (y‐y^{⋀})$$ (3)

The long-term and short-term memory network, based on the simple cyclic neural network, specializes in cyclic information transmission by introducing a new internal state c_t_∈R^D^. At the same time, it outputs information nonlinearly to the external state h_t_∈R^D^ of the hidden layer, which solves the problem of gradient explosion or disappearance. The internal state c_t_ is calculated by the following Formulas (4)–(5).


$$c_{t}=f_{t}⊙c_{t‐1}+i_{t}⊙c_{t}^{⋀}$$ (4)

$$h_{t}=o_{t}⊙\tanh(c_{t})$$ (5)

Among them, the forget gate f_t_∈[0,1]^D^, the input gate i_t_∈[0,1]^D^, and the output gate o_t_∈[0,1]^D^ are three gates to control the information transmission path. ⊙ is the product of vector elements, the internal state c_t−1_ is the memory unit at the previous moment, and the candidate state $c_{t}^{⋀}\in R^{D}$ is the candidate state obtained through a nonlinear function:

$$c_{t}^{⋀}=\tanh(W_{c}x_{t}+U_{c}h_{t‐1}+b_{c})$$ (6)

**Fig 2:**
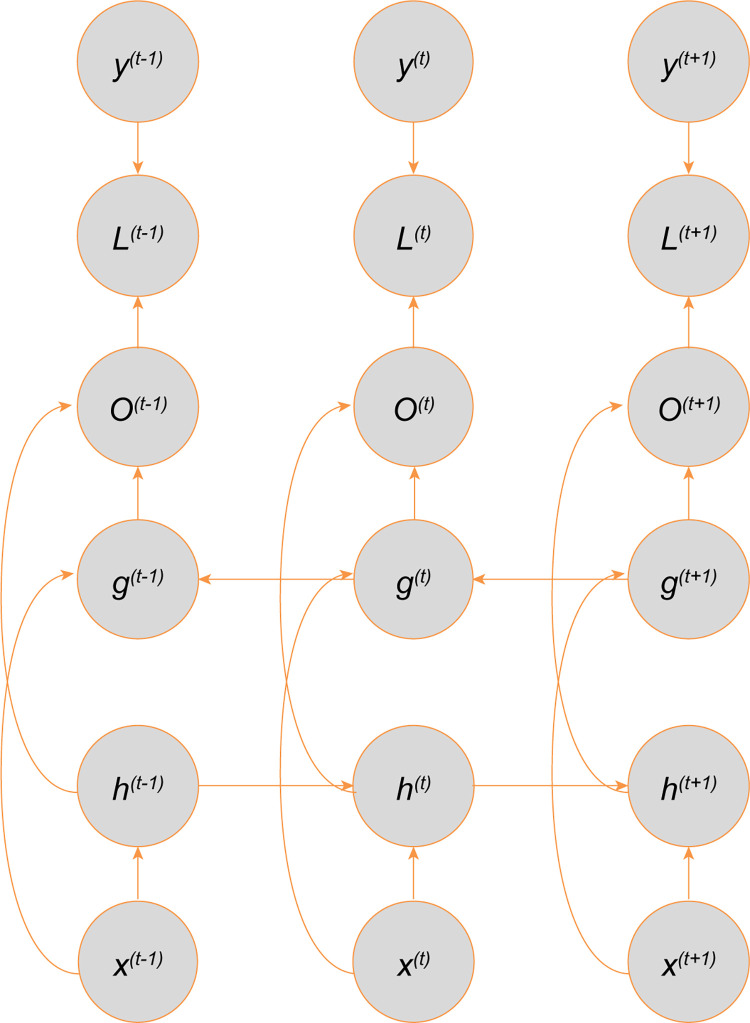
The computational graph to compute the training loss of the bidirectional recurrent network.

The bidirectional recurrent neural network (Bi-RNN) combines the RNN that moves forward and the RNN that moves backward in the time series. The basic Bi-RNN training loss expansion calculation diagram is shown in Fig 2. The hidden state h recursively propagates to the left in the time sequence, the hidden state g recursively propagates to the right in the time sequence, and their inputs are the same.

The hidden state at t is defined as $h_{t}^{\left(1\right)}$ and $g_{t}^{\left(1\right)}$ (see calculation expressions (7)–(9)), where o_*t*_ is the vector concatenation of $h_{t}^{\left(1\right)}$ and $g_{t}^{\left(2\right)}$.


$$h_{t}^{\left(1\right)}=f(U^{1}h_{\left(t‐1\right)}^{\left(1\right)}+W^{\left(1\right)}x_{t}+b^{\left(1\right)})$$ (7)

$$g_{t}^{\left(2\right)}=f(U^{2}g_{\left(t+1\right)}^{\left(1\right)}+W^{\left(2\right)}x_{t}+b^{\left(2\right)})$$ (8)

$$o_{t}=h_{t}^{\left(1\right)}⨁g_{t}^{\left(2\right)}$$ (9)

### Construction of the tailings pond monitoring and early warning system

#### System structure

**Fig 3:**
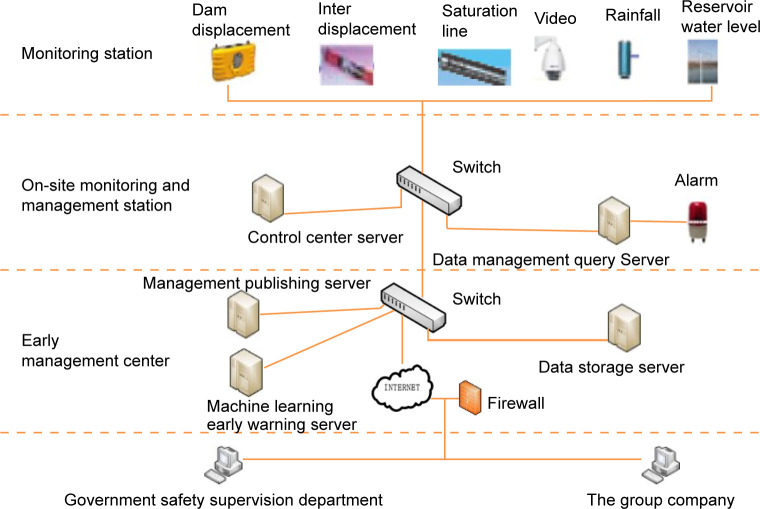
Monitoring and early warning system architecture.

The system structure is shown in Fig 3, which adopts a three-level structure that includes a monitoring station, a monitoring management station and a prediction management center. As the first level of the overall architecture, the monitoring station is used to obtain the data of monitoring points in real time. The on-site monitoring and management station is the second level of the overall architecture, which is used for data collection, display, query, fault alarm, etc. The prediction management center station is the third level of the overall architecture and is used for 3D display, collection, storage, management, analysis, early warning and remote network release of the data.

Global navigation satellite system (GNSS) ground receiving sensors are used for dam displacement monitoring. According to the requirements of the code, a monitoring section is set with a section spacing of 100~300 m, and multiple monitoring sections are set in the dam body. There are multiple monitoring points in each section, a monitoring reference point is set in the stable area near the duty room, and a reference point is set in the stable area at the dam tail. GNSS data processing software is deployed in the control center server, and all monitoring points and reference points of the tailings pond transmit GNSS signals received in real time with the control center through the network. The GNSS data processing software denoises and solves the position information of each monitoring point in real time according to the model and analyzes it by associating the initial coordinates, thus obtaining the displacement variation of each monitoring point. The main functions of the GNSS data solution software are the remote management of GNSS receivers at monitoring points and control points, real-time and on-time GNSS raw data analysis and processing, independent ring network adjustment and data management.

Internal displacement monitoring and internal displacement monitoring points should be deployed in combination with surface displacement, and monitoring sections should be set at the dam crest at the initial stage of the tailings pond. Each section is provided with a plurality of monitoring vertical lines, and each vertical line is provided with a plurality of sensors to monitor the displacement in the downstream direction of the dam body axis.

*Infiltration line monitoring*. The infiltration line is monitored by using the intelligent vibrating string osmometer built in the piezometer. The osmometer sensor transmits data to the data acquisition unit through special hydraulic cables, converges them with other data through communication cables, and finally transmits all data to the monitoring center server through an industrial network. For reservoir water level monitoring, a radar level gauge is adopted, and monitoring points are set on the drainage wells in the reservoir area. The radar level gauge moves to monitor the change in the reservoir water level. For rainfall monitoring, a grid rain gauge with a heating module is adopted, and monitoring points are set at the duty room on site. Video surveillance deployment, using high-definition infrared network cameras, sets up monitoring points near the duty room of the reservoir area, the top of the initial dam, the drainage well, etc., to meet the night vision and key area monitoring requirements.

#### Early warning management center

The early warning management center of the system is deployed in the mine management center, centrally manages the monitoring of the tailings pond, sets up the storage server, machine learning early warning system and data management publishing server, and is responsible for and realizes the inquiry, analysis and early warning of the monitoring system. According to the monitoring items, it has good stability and expandability, meets the needs of mine sites and remote management, and can realize networking with relevant government supervision departments.

**Fig 4:**
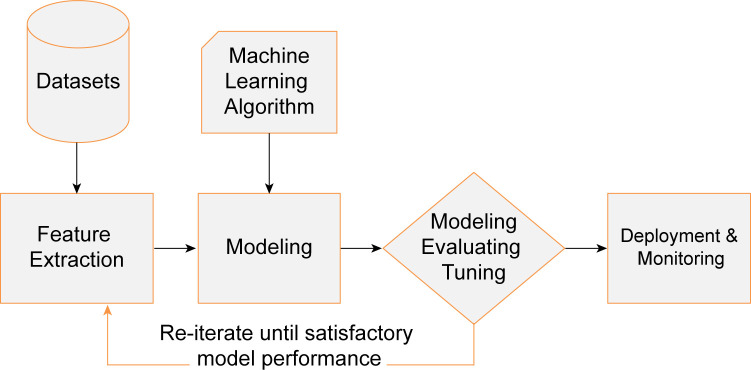
Machine learning process.

**Fig 5:**
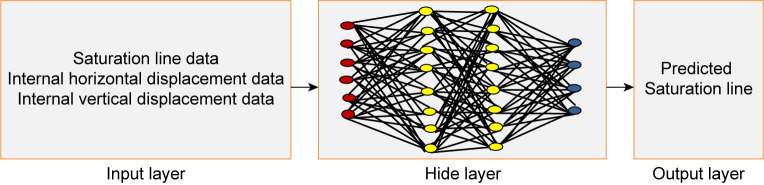
Basic structure of the model.

The goal of the machine learning server is to automate the decision-making of tasks. The learning process is shown in Fig 4. Feature extraction involves extracting important features or attributes from the original data or creating new features from existing features. Modeling involves providing data features to machine learning methods or algorithms and training them, aiming at the evaluation index of the loss function, reducing errors and summarizing expressions learned from the data. Model evaluation and adjustment involve evaluation and testing on the validation dataset and gradual optimization to obtain the optimal model. The basic structure of the model is shown in Fig 5. The input data are the time series data of the infiltration line and internal displacement, the output layer is the prediction data of the infiltration line, and the hidden layer performs deep learning according to the algorithm. For deployment and monitoring, the selected model is deployed in production and continuously monitored according to its prediction and results.

## Experiment

### Experimental environment

Model training machine hardware configuration: 8-core AMD Ryzen7 2.00 GHz processor and 16 GB RAM. Software: Python 3.8.5 and TensorFlow 2.3.0.

### Experimental design

The goal of the experiment is to answer two questions:

1. What is the loss difference of the model when different optimization algorithms are utilized?
2. How does the multilayer perceptron model compare with a model based on a bidirectional recurrent long short-term memory network?

**Fig 6:**
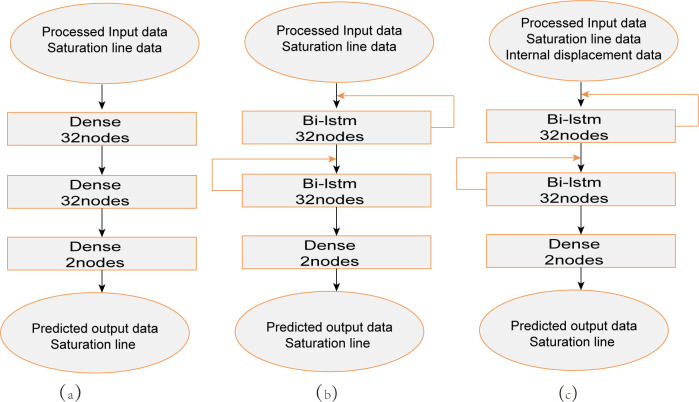
Experimental Model Structure (a) Multi-Layer Perceptron Model, (b) Univariate Input Model, and (c) Multivariable Input Model.

To answer the first question, we use algorithms such as stochastic gradient descent (SGD), adaptive gradient (AdaGrad), root mean square prop (RMSprop), and adaptive moment estimation (Adam). For the second question, the multilayer perceptron model and the model based on the bidirectional recurrent long-short memory network are compared, the model structure diagram is shown in Fig 6. This model contains three layers. The multilayer perceptron model consists of all fully connected layers, with 32 nodes in the first layer, 32 nodes in the second layer, and 2 nodes in the third layer. Based on the bidirectional cyclic long-short memory network model, the first and second layers are bidirectional cyclic long-short memory network layers, each layer with 32 nodes, and the third layer is a fully connected layer with 2 nodes.

### Model evaluation index

The root mean square error (RMSE) was chosen as the evaluation index of model performance. The RMSE is expressed as follows:

RMSE=1n∑i=1n(yi−yi^)2 (10)

### Data preparation

The research data come from the monitoring system database of a metal mine tailings pond in western China. The monitoring equipment for the saturation line of the metal mine tailings pond adopts an intelligent vibrating wire sensor, and the internal displacement monitoring equipment adopts an intelligent inclinometer. According to the monitoring design of the tailings pond, the monitoring system of the whole tailings pond is composed of seven cross-sections, each of which has 3–4 monitoring points of the saturation line and one internal displacement monitoring point, and each monitoring point has different buried depths of the intelligent vibrating wire sensors and intelligent inclinometers.

**Fig 7:**
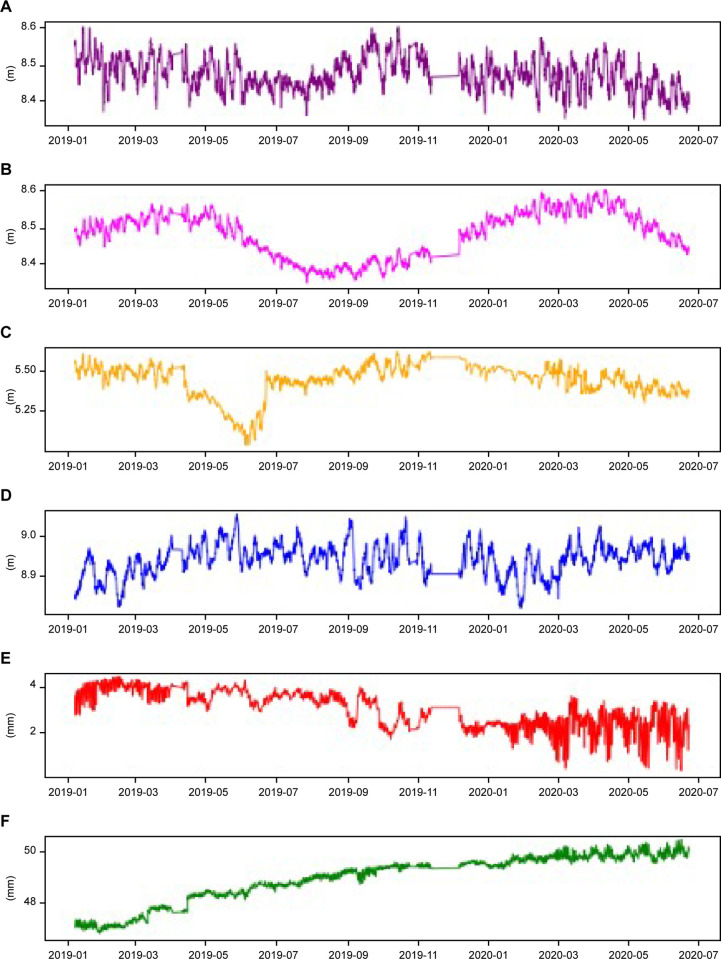
Saturation line height and internal displacement over time.

In this study, the state of the infiltration line over the next six hours is predicted, mainly using the databases of four monitoring points of the infiltration line in cross-section 1 and the databases of the horizontal internal displacement and vertical internal displacement of adjacent internal displacement monitoring points, which are named DataSetI, DataSetII, DataSetIII, DataSetIV, DataSetV and DataSetVI. The monitoring data are collected every three hours from January 2019 to June 2020. There are 8 monitoring data points every day and 3,850 records in each dataset. The curve of the data collected by the monitoring points with time is shown in Fig 7, and some original data are shown in Table 1.

**Table 1:** Part of the original saturated line and internal displacement data.

Acquisition time	DataSetI (m)	DataSetII (m)	DataSetIII (m)	DataSetIV (m)	DataSetV (mm)	DataSetVI (mm)
2019-01-08 01:06:00	8.5534	7.6418	5.5572	8.8431	3.5777	47.0169
2019-01-08 04:06:00	8.5633	7.6418	5.5549	8.8508	3.2718	47.0885
2019-01-08 07:06:00	8.5668	7.6443	5.5450	8.8525	3.0608	47.1234
2019-01-08 10:06:00	8.5590	7.6350	5.5519	8.8525	2.8494	47.2027

To improve the convergence speed and accuracy of the model, the collected data are normalized. The normalization method adopted is the MinMaxScalar method, and the calculation formula is as follows:

$$X_{\mathrm{std}}=\frac{X‐X.\min}{X.\max‐X.\min}$$ (11)

$$X_{\mathrm{scaled}}=X_{\mathrm{std}}*(\max‐\min)+\min$$ (12)

where X is the original saturation line data or internal displacement data; x.min and x.max are the maximum and minimum, respectively; max and min are the normalized characteristic ranges, which are 1 and 0 by default; and X_scaled_ is the normalized data.

### Model training

The model parameters depend on the input layer, hidden layer and output layer of the model. The dimension of the input layer depends on the feature dimension of the training set. In this study, there are one-dimensional and three-dimensional time series data, which correspond to the infiltration line, internal horizontal displacement and internal vertical displacement time series data. There are many neurons in the hidden layer. These neurons transform the input of the previous layer and then use the activation function to activate it and output it to the next layer. The output layer is the predicted target result.

Although deep learning is very powerful, it requires different optimization methods, such as stochastic gradient descent (SGD), adaptive gradient (AdaGrad), root mean square prop (RMSprop), and adaptive moment estimation (Adam). When the input sequence x_1:T_ = (x_1_,…,x_T_) of length T and the label sequence y_1:T_ = (y_1_,…y_T_) constitute the training sample (x, y), there is label supervision information y_t_ at time t, and the loss function is defined as:

$$L_{t}=L(y_{t},g(h_{t}))$$ (13)

L is the differentiable loss function, g(h_t_) is the output at time t, and the loss function of the entire sequence is:

$$L=\sum_{t=1}^{t}L_{T}$$ (14)

The mean squared error (MSE) loss function can be expressed mathematically as:

$$L_{\mathrm{mse}}={\left(y^{t}‐g(h_{t})\right)}^{2}$$ (15)

The gradient of the loss function L of the entire sequence with respect to the weight parameter U is the sum of the partial derivatives of the loss L_t_ with respect to the parameter U at each moment. The expression is as follows:

$$\frac{\partial L}{\partial U}=\sum_{t=1}^{T}\frac{\partial L_{t}}{\partial U}$$ (16)

Calculate the partial derivative $\frac{\partial L_{t}}{\partial U}$ because the weight parameter U and the net input of the hidden layer at each time k (1≤k≤t) are:

$$z_{k}=Uh_{k‐1}+Wx_{k}+b$$ (17)

Therefore, the gradient of the loss function L_t_ with respect to the parameter u_*ij*_ at time t is:

$$\frac{\partial L_{t}}{\partial u_{\mathrm{ij}}}=\sum_{k=1}^{t}\frac{\partial^{+}z_{k}}{\partial u_{\mathrm{ij}}}\frac{\partial L_{t}}{\partial z_{k}}$$ (18)

where $\frac{\partial^{+}z_{k}}{\partial u_{ij}}=[0,\ldots ,[h_{k-1}],\ldots ,0]$ and the error term $\delta_{t,k}=\frac{\partial L_{t}}{\partial z_{k}}$ is defined as the derivative of the loss at time t with respect to the net input *z*_*k*_ of the hidden neural layer at time k. Then, when 1≤k≤t,

$$\delta_{t,k}=\frac{\partial L_{t}}{\partial z_{k}}=\frac{\partial h_{k}}{\partial z_{k}}=\frac{\partial z_{k+1}}{\partial h_{k}}\frac{\partial L_{t}}{\partial z_{k+1}}$$ (19)

Substituting Formulas (18) and (17) into Formula (16) yields a matrix of the form:

$$\frac{\partial L_{t}}{\partial U}=\sum_{k=1}^{t}\delta_{t,k}h_{k-1}^{T}$$ (20)

The process of model training is to apply an optimization algorithm, iterate the model parameters, gradually improve the loss of the model and index, minimize the loss function, and terminate the model iteration to obtain the optimal parameters learned from the model training. The model training is time consuming, sometimes requiring hours or even weeks. The efficiency of model training is related to the advantages and disadvantages of the optimization algorithm. Understanding the optimization algorithm is beneficial to targeted model parameter adjustment, which makes the model perform better. The training models in this experiment include a multivariate input infiltration line prediction model and a univariate input infiltration line prediction model.

**Algorithm 1. Back-propagation algorithm stochastic gradient descent optimization training.**

Input: training set $D={\left(x^{\left(n\right)}y^{\left(n\right)}\right)}_{n=1}^{N}$, validation set V, learning rate α, regularization system λ, number of network layers L, number of neurons Ml, 1≤l≤L.

1: Random initialization W, b;

2: Repeat

3: Randomly reorder the samples in the training set;

4: For n = 1…N do

5: Select samples (x(n),y(n)) from training set D;

6: Feed-forward calculate the net input z(l) and activation value a(l) of each layer until reaching the last layer;

7: Inversely calculate the error δ(l) of each layer;

8: ∀l,∂L(y(n),y^(t))∂w(l)=δ(l)(α(l−1))T;

9: ∀l,∂L(y(n),y^(t))∂b(l)=σ(l);

10: $W^{\left(l\right)}\leftarrow W^{\left(l\right)}-\alpha (\sigma^{\left(l\right)}{\left(a^{l-1}\right)}^{T}+\lambda W^{\left(l\right)})$;

11: b^(l)^←b^(l)^−αδ^(l)^;

12: End

13: Until the error rate of the deep Bi-LSTM network model on verification set V no longer decreases;

Output: W, b

The parameter update difference Δ*θ*_*t*_ of the adaptive moment estimation algorithm (Adam) is calculated using the Formulas (21)–(23), where α is the learning rate, β_1_ and *β*_2_ are the decay rates, ε is a very small constant to maintain numerical stability, and Mt^ is the modified first-order moment bias, and Gt^ is the corrected second moment bias.


Mt^=Mt1−β1t (21)

Gt^=Gt1−β2t (22)

Δθt=−αGt^+ϵMt^ (23)

The univariate input infiltration line prediction model uses DataSetII for training the input data. For example, Algorithm 1 depicts SGD random gradient descent optimization training. Train the model 10,000 times.

The training input data of the multivariate input model adopted include DataSetII, DataSetV, and DataSetVI (i.e., the input data are the saturation line, internal horizontal displacement and internal vertical displacement). The univariate input infiltration line prediction model and the multivariate input infiltration line prediction model adopted are the Adam optimization method and the same evaluation standard. The model was trained 10,000 times.

## Results and discussion

The univariate input infiltration line prediction model of the deep learning bidirectional cyclic long and short memory network is used to perform prediction on DataSetI, DataSetII, DataSetIII and DataSetV, as shown in Table 2, where the prediction root mean square error (RMSE) is used to compare performance. The Adam optimization algorithm has the lowest RMSE (approximately 0.046) among these optimization methods, while the RMSprop optimization algorithm has the highest prediction RMSE (approximately 0.119).

**Table 2:** Prediction RMSE of different optimized methods.

Dataset	SGD	AdaGrad	RMSprop	Adam
I	0.04711	0.04738	0.04743	0.04688
II	0.04726	0.04844	0.04849	0.04599
III	0.07602	0.10724	0.10762	0.06145
IV	0.10902	0.11966	0.11983	0.09966

As shown in Table 3, the multivariate input infiltration line prediction model and multilayer perceptron model carry out predictions on DataSetI, DataSetII, DataSetIII and DataSetV. Comparing the loss between the multilayer perceptron model, multivariate input model and univariate input model, the multivariate input infiltration line model is slight worse. The RMSE of prediction is basically satisfactory and can provide some decision support.

**Table 3:** Prediction RMSE of different models.

Model	DataSetI	DataSetII	DataSetIII	DataSetIV
Multilayer perceptron model	0.04688	0.04658	0.07486	0.10611
Univariate input model	0.04688	0.04599	0.06145	0.09966
Multivariable input model	0.04692	0.04871	0.10855	0.11955

This article fuses deep learning technology in the construction of a tailings pond early warning system. At present, many tailings pond early warning systems compare with the threshold after real-time data collection for early warning, which is somewhat different from the data-driven early warning method. The early warning indicators of tailings ponds include dam body displacement, internal displacement, infiltration line, reservoir water level, rainfall, and infiltration line. The prediction index of the early warning model fused with deep learning in this paper is the infiltration line. The experimental data adopted are the same cross-sectional infiltration line monitoring point data and adjacent internal displacement data. Although there are certain limitations, the fusion of multisource data of the infiltration line and internal displacement is realized, and the trained model can be migrated to related cross-sections to improve the efficiency of model training as a whole.

## Conclusions

In this paper, a method for constructing the monitoring and early warning system of tailings reservoirs that includes the infiltration line, dam displacement, internal displacement, reservoir water level, rainfall, video, etc., is introduced, and an infiltration line prediction model of a bidirectional recurrent long and short memory network is proposed, which provides technical support for the design and daily management of monitoring and early warning systems of tailings reservoirs.

The tailings pond monitoring and early warning system offers more real-time response and intelligence, and the data-driven tailings pond risk early warning method has certain applicability. In the early warning of issues related to the tailings reservoir infiltration line, comparing the multilayer perceptron model, the univariate input model, and the multivariate input model, their RMSEs are 0.10611, 0.09966, and 0.11955, respectively. The data-driven early warning of tailings pond risk, integrating monitoring indicators such as dam body displacement, internal displacement, the infiltration line, the reservoir water level, and rainfall, is conducive to further risk evaluation.

## Supporting information

S1 Dataset (CSV)

S1 File (PDF)
