# Supplementary material for: Monitoring and early warning of a metal mine tailings pond based on a deep learning bidirectional recurrent long and short memory network
Source: PLoS One. 2022 Oct 13;17(10):e0273073. doi: 10.1371/journal.pone.0273073 (PMC9562152; doi:10.1371/journal.pone.0273073)
Supplement: S1 File — (PDF) [file pone.0273073.s002.pdf]

Graphic Abstract

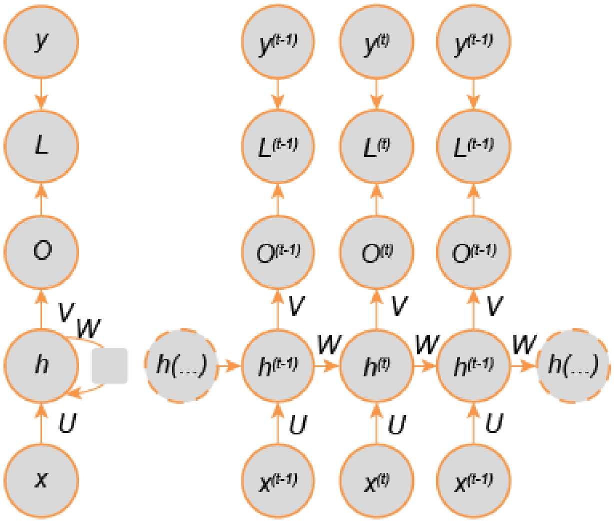

Fig 1. The Computational Graph to Compute the Training Loss of the Recurrent Network.

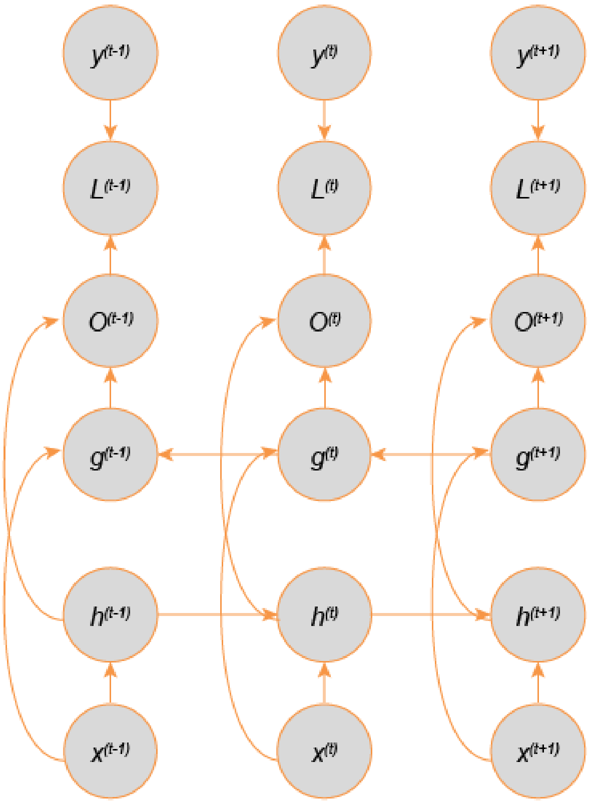

Fig 2. The Computational Graph to Compute the Training Loss of the Bidirectional Recurrent Network.

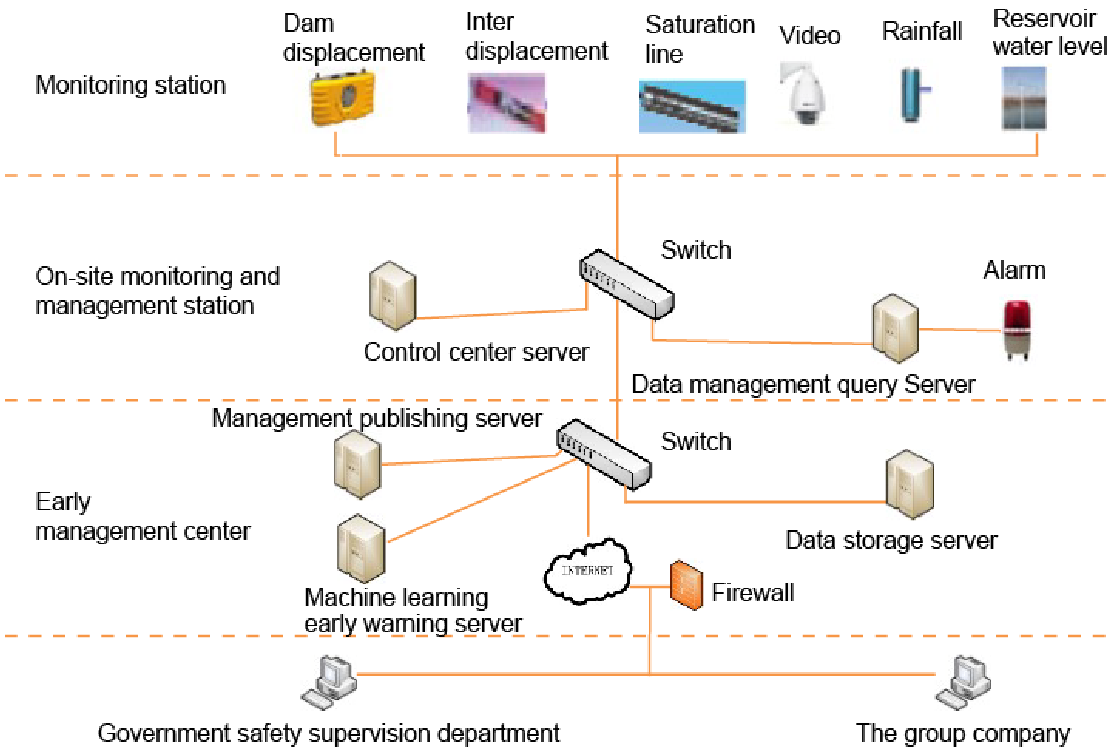

Fig 3. Monitoring and Early Warning System Architecture.

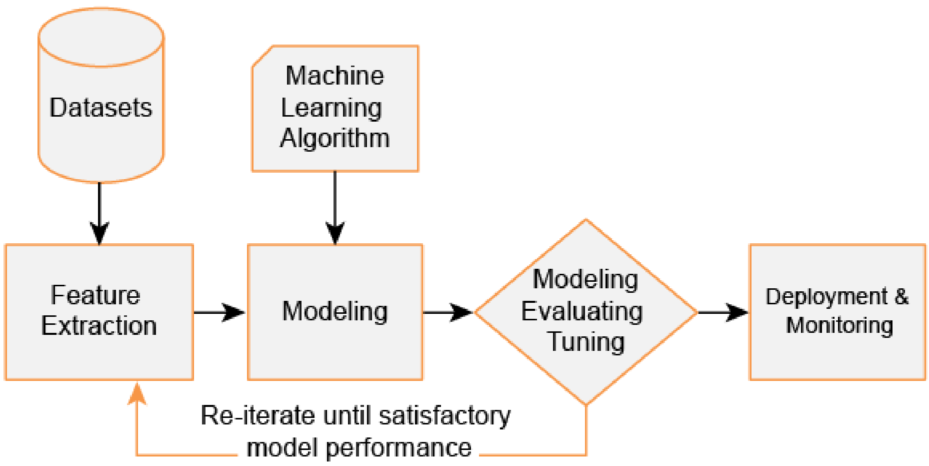

Fig 4. Machine Learning Process.

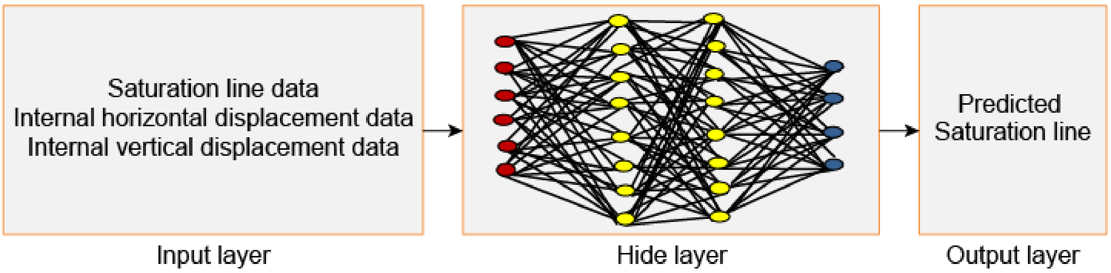

Fig 5. The Basic Structure of the Model.

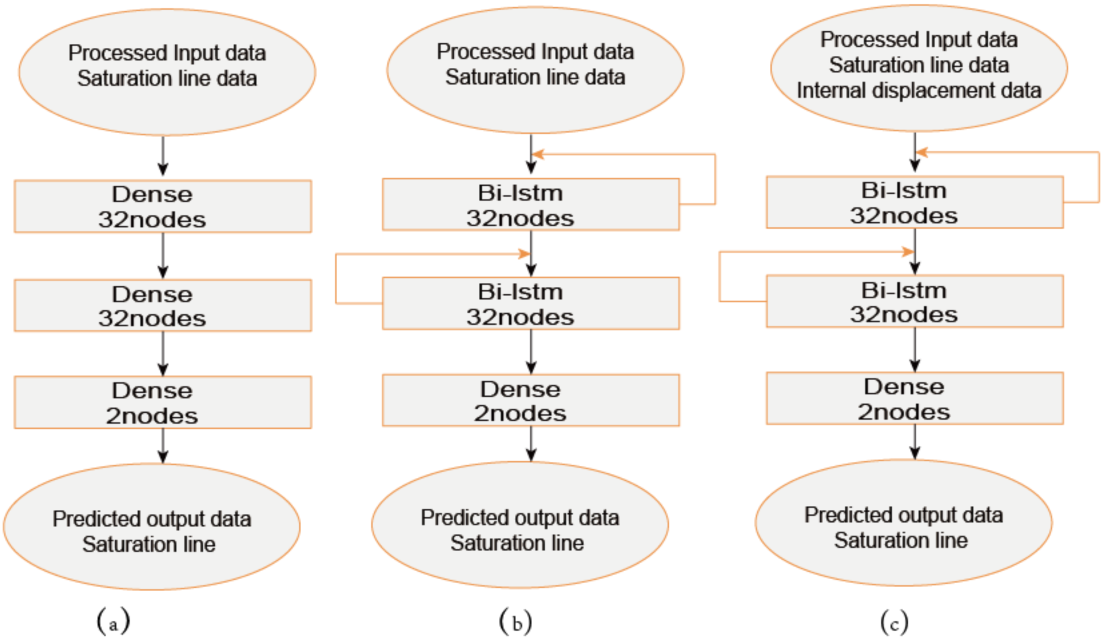

Fig 6. Experimental model structure (a) multi-layer perceptron model, (b) univariate input model, and (c) multivariable input model.

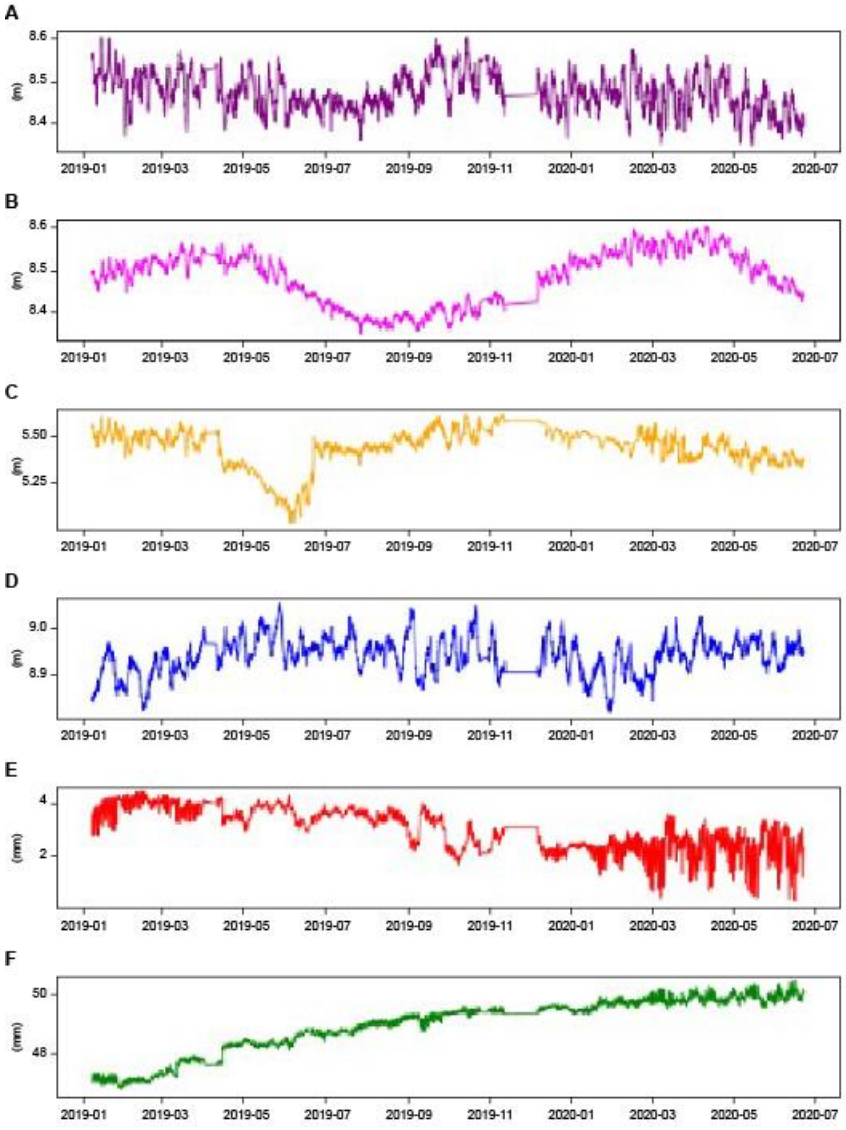

Fig 7. Saturation Line Height and Internal Displacement Over Time.
